# Enzymatic hydrolysis of gluten proteins changes the dose-response of oral sensitization, but not the gluten epitopes

**DOI:** 10.1186/2045-7022-3-S3-O17

**Published:** 2013-07-25

**Authors:** S Kroghsbo, NB Andersen, TF Rasmussen, S Jacobsen, CB Madsen

**Affiliations:** 1 Division of Toxicology and Risk Assessment, DTU National Food Institute, Soborg, Denmark; 2 Enzyme and Protein Chemistry, DTU Systems Biology, Lyngby, Denmark

## Background

Wheat gluten proteins are very complex proteins containing hundreds of components present as monomers, oligomers and polymers that by definition are not soluble in water. To improve utilization, gluten proteins can be hydrolyzed by enzymes or acid. This increases solubility and provide proteins with new functional properties. During the last decade, cases of severe allergic reaction to hydrolyzed wheat proteins have been reported in subjects tolerant to wheat.

## Methods

Brown Norway rats bred and kept on a gluten-free diet for at least three generations were sensitized either orally or intraperitoneally (i.p.) with two different commercial gluten products; unmodified (U-gluten) and enzymatic hydrolyzed gluten (EH-gluten). No adjuvant was used for sensitization. For oral sensitization rats were dosed by daily gavage for 35 days with 0.2, 2 or 20 mg of gluten protein suspended in PBS. For i.p. sensitization rats were immunized three times two weeks apart with 200 µg of gluten protein in PBS. Sera obtained at sacrifice were analyzed by ELISA for levels of specific IgG1 and IgE and for IgG1-binding capacity by inhibition ELISA. Biological functionality of specific IgE was examined by rat basophilic leukemia cell (RBL) assay.

## Results

Enzymatic hydrolysis significantly increased solubility of gluten proteins. Regardless of the route of dosing, U-gluten and EH-gluten were found to have sensitizing capacity. Sensitization by the i.p. route induced a strong specific IgG1 and IgE response in all animals. No difference in levels or functionality of specific IgG1 and IgE were found between i.p. immunized groups. A clear dose-response in levels of specific IgG1 and IgE was found for rats dosed orally with EH-gluten while U-gluten induced comparable antibody levels and number of responders irrespective of the dose used for oral sensitization. All doses of U-gluten gave a higher response than low dose EH-gluten and a lower response than high dose EH-gluten. As for the i.p. studies, IgG1-binding was not influenced by the gluten product used for oral sensitization.

## Conclusion

Our results indicate that epitopes are not lost and new epitopes are not formed or disclosed during enzymatic hydrolysis. While enzymatic hydrolysis does not change sensitization by the i.p. route, dose-response was clearly changed when dosed orally. As EH-gluten was a less potent sensitizer at low dose our results indicate that enzymatic hydrolysis reduces sensitizing capacity despite the fact that epitopes seem unharmed.

## Disclosure of interest

None declared.

